# FACTORS ASSOCIATED WITH THE PREVALENCE AND INTENSITY OF DENTAL PAIN IN CHILDREN IN THE MUNICIPALITIES OF THE CAMPINAS REGION, SÃO PAULO

**DOI:** 10.1590/1984-0462/;2017;35;3;00001

**Published:** 2017-07-31

**Authors:** Renata Cristina Guskuma, Vinícius Aguiar Lages, Maylu Botta Hafner, Maria Paula Maciel Rando-Meirelles, Silvia Cypriano, Maria da Luz Rosário de Sousa, Marília Jesus Batista

**Affiliations:** a Faculdade de Odontologia de Piracicaba, Universidade Estadual de Campinas, Piracicaba, SP, Brasil.; b Pontifícia Universidade Católica de Campinas, Campinas, SP, Brasil.

**Keywords:** Dental pain, Children, Absenteeism

## Abstract

**Objective:**

The aim of this study was to evaluate the prevalence and intensity of dental pain in children according to size of municipality, associated factors and absenteeism.

**Methods:**

The sample consisted of children aged 12 years old from public and private schools drawn from eight cities in the region of Campinas (SP). A questionnaire was applied to obtain dental pain, demographic, socioeconomic data, and a clinical examination was carried out to evaluate the experience of having a cavity. The outcome for the logistic regression analysis was having pain and the outcome for the negative log-binomial regression was the intensity of pain. The significance level was 5%.

**Results:**

The sample consisted of 1,233 children, and 16.7% reported pain in the last six months. Dental pain was the cause of 46.4% of school absenteeism during this period. The prevalence of pain was lower among households with high income (*p*=0.023) and higher among nonwhites (*p*=0.027). Pain intensity was lower in medium-sized cities (*p*=0.02) and small cities (*p*=0.004), and higher in children whose parents had a lower educational level (*p*=0.003), children who sought out a dentist for the pain (*p*=0.04) and who had untreated cavities (*p*=0.04).

**Conclusions:**

The prevalence and intensity of dental pain in children aged under 12 are related to socioeconomic aspects of the family, such as low-income and parents with a low level of education, which impact daily activities as seen through school absenteeism. Pain intensity was lower in medium and small cities. Oral health promotion strategies in this age group should be encouraged to avoid dental pain.

## RESUMO

**Objetivo:**

Avaliar a prevalência e a intensidade de odontalgia em crianças segundo porte populacional do município, fatores associados e absenteísmo.

**Métodos:**

A amostra constituiu-se de crianças de 12 anos provenientes de escolas públicas e privadas, sorteadas em oito cidades da região de Campinas (SP). Foi aplicado um questionário com dados de odontalgia, demográficos, socioeconômicos bem como foi realizado exame clínico para avaliar a experiência de cárie. Ter dor foi o desfecho para análise de regressão logística e a intensidade da dor para a regressão log-binomial negativa. Adotou-se nível de significância de 5%.

**Resultados:**

A amostra foi constituída de 1.233 crianças, sendo que 16,7% relataram dor nos últimos seis meses. A odontalgia foi causa de 46,4% do absenteísmo escolar nesse período. A prevalência de dor foi menor entre os de alta renda familiar (*p*=0,023) e maior entre não brancos (*p*=0,027). A intensidade da dor foi menor nos municípios de médio (*p*=0,02) e pequeno porte (*p*=0,004) e maior nas crianças cujos pais tinham menor nível de escolaridade (*p*=0,003), que procuraram o dentista por dor (*p*=0,04) e que apresentavam cárie não tratada (*p*=0,04).

**Conclusões:**

A prevalência e a intensidade da dor de dente em crianças de 12 anos estão relacionadas com aspectos socioeconômicos da família, como baixa renda e menor escolaridade dos pais, e causam impacto na atividade diária por meio do absenteísmo escolar. A intensidade foi menor em municípios de médio e pequeno porte. Estratégias de promoção de saúde bucal nessa faixa etária devem ser estimuladas para evitar a odontalgia.

## INTRODUCTION 

The International Association for the Study of Pain (IASP) conceives of pain as an unpleasant sensory and emotional experience that promotes changes in a person’s behavior, often hindering the normal performance of their daily activities.^1^ This complex phenomenon is a relatively common symptom in dental diseases, and can have a negative impact on the quality of life of people, resulting in absenteeism.^2^ ^,^ ^3^ The main cause of dental pain among children is cavaties^2^, but pain is also associated with erosion, trauma and exfoliation of deciduous teeth.^4^

The prevalence of dental pain among schoolchildren varies widely between countries: from 9% in Japan^5^ to 40% in England.^6^ In Brazil, this prevalence is also variable, with rates ranging between 11 and 39%.^7^ ^,^ ^8^ ^,^ ^9^ Dental cavities and their after-effects are often associated with emergency visits to the dentist. There is also evidence that prevalence is higher in populations with less access to dental services, such as children of lower social classes and populations in which dental cavities are not treated. Thus, children with less access to care are more likely to have extensive, and therefore, more painful carious lesions.^4^ ^,^ ^7^ ^,^ ^9^ ^,^ ^10^

Oral problems can cause nutritional deficiencies, impaired aesthetics, phonation, chewing and swallowing as well as low self-esteem.^9^ ^,^ ^10^ In addition, children who have dental problems and do not have access to dental services are more prone to absenteeism, which affects school performance.^11^ Characteristics such as race, parents’ low schooling and low socioeconomic status were associated with worse school performance in children.^10^ ^12^ Recent studies^13^ ^,^ ^14^ point to contextual variables associated with dental pain in preschool children, such as low levels in the Human Development Index (HDI), showing that one’s environment may reflect inequalities in the development of cavities.

The search for a better understanding of this phenomenon, as well as its reduction or interruption, is of great importance for science and society. Thus, this issue must be addressed during the planning of health promotion actions. This article is different in that it addresses, in addition to individual socioeconomic factors, the possible effect of municipality population size on dental pain. The objective of this study was to evaluate the prevalence and intensity of pain in schoolchildren aged 12 years according to the municipality’s population size, associated factors and absenteeism.

## METHOD

This was a cross-sectional study carried out with 12 year-old schoolchildren, which is an age established internationally as a basic parameter in studies of oral health with permanent dentition in children. The research was approved by the Research Ethics Committee of the School of Dentistry of Piracicaba (FOP/Unicamp), under protocol 105/2010. A Free and Informed Consent Form (FICE) was sent to the parents of the students selected to participate in the survey, which was signed after they agreed to participate.

This study comes from the secondary data of the Oral Health Epidemiological Survey, from the Regional Health Board (DRS) VII 2010, entitled “Oral Health Conditions in Municipalities located in the region of Campinas” and carried out by the State Health Department of Sao Paulo. The students evaluated attended public and private schools selected in these cities, which were classified according to size based on data from the Brazilian Institute of Geography and Statistics (IBGE).^15^

The sample was systematic and probabilistic, in order to represent each of the eight municipalities. Sample selection in the municipalities took place in two stages. In the first stage, the Primary Sampling Units (PSU) were the schools and, in the second stage, the school children. Thus, 20 schools were selected in each municipality, and were systematically organized according to the number of students. Subsequently, 12 year-old students were selected. When conducting the exams, the sampling without replacement rule was adopted.

The sample calculation for each municipality that composed this sample is in accordance with data from the Oral Health Conditions of the State of São Paulo from 2002.^16^ A 95% confidence interval (95%CI), 20% accuracy, and a design effect (deff) of 2 was adopted. 20% was added to the total to compensate for eventual losses and refusals. The final sample size was: Valinhos, 230 children; Lindoia, 263; Vineyard, 301; Amparo, 199; Águas de Lindoia, 184; Indaiatuba, 184; Monte Alegre, 184; and Monte Mor, 184, totaling 1,729 children.

Team calibration, which was composed of four examiners (dental surgeons) per municipality, was performed by the same reference examiner through theoretical discussions and practical activities, simulating the different conditions and situations that the professionals would encounter during the 20 hours of practical work. The percentage of inter-examiner and intra-examiner agreement was from 95% and 98% to 100%, respectively, for the present and past experiences of cavity attacks to permanent dentition, which is estimated by the Decay-missing-filled teeth index (DMFT). For the calculation of DMFT, the sum of the number of teeth with carious lesions (component C), missing teeth (component P) and restored teeth (component O) is calculated. The values of this index correspond to the following degrees of severity: very low (0.0-1.1), low (1.2-2.6), moderate (2.7-4.4), high (4.5 -6.5) and very high (≥6.6).^17^ Data was recorded by previously trained scorers in individual records. Kappa ranged from 0.91 to 1.

Data was collected in clinical examinations in the schools selected as well as from a structured questionnaire, which was sent to the parents of the schoolchildren selected at the school. Dental examinations followed the methodology proposed by the World Health Organization (WHO).^17^ A flat mouth mirror and a spherical periodontal probe were used under natural light, with the examiner and the examined person seated. In addition to the cavity being evaluated by the DMFT index at the time of examination, dental treatment needs were also verified according to the criteria proposed by the WHO.

The children responded to a pain report in an interview with the dental surgeon, while their parents or guardians filled out the questionnaire. To evaluate pain intensity, a numerical rating scale (NRS)^18^ was used for the self-perception of pain with values ranging ​​from 1 to 10, in which one was the lowest pain intensity, and ten, the highest. The questionnaire addressed issues related to the use of dental services, toothache in the last six months, school absenteeism reported by parents as a consequence of this pain in the last six months, as well as demographic and socioeconomic aspects.

In this study, the outcome was the reporting of pain report (yes or no) in the last six months and the intensity of pain (ranging from 1 to 10). The independent variables analyzed were: population of the cities, socioeconomic aspects (income and educational level of parents), demographic aspects (gender and race), use of dental services (visit to the dentist, reason for consultation and frequency) and presence of cavities.

Independent variables were grouped into categories and recoded. The population size of cities was divided into three classes according to IBGE criteria:^15^ small (up to 50,000 inhabitants), medium (50,001 to 100,000 inhabitants) and large (over 100,001 inhabitants). The variable “visit to the dentist” was dichotomized into yes and no; “time since last visit to the dentist” was divided into more than one year and less than one year; “use of a dental service” was divided into public, private and insurance; “parental schooling” was divided into more or less than eight years; “reason to go to the dentist” was divided into pain, treatment and routine; “race” was divided into white and nonwhite; And “family income” was divided in up to R$ 500.00, between R$ 501.00 and R$ 1.500,00 and more than R$ 1,501.00.

**Figure 1:**
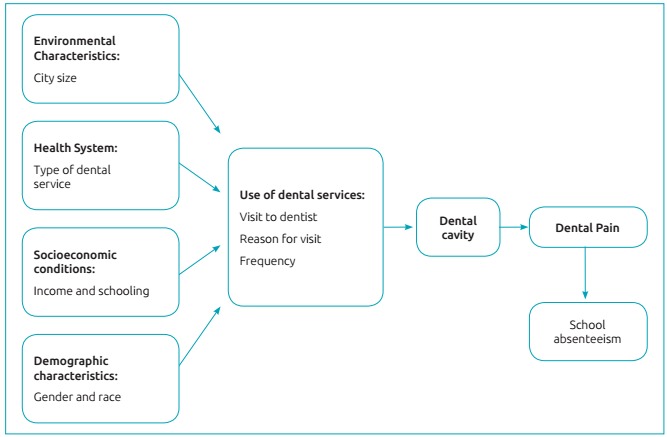
Theoretical conceptual model used for the study.

For the analysis of the results, SPSS was used. Descriptive statistical analyses of the variables were performed. The prevalence of pain (pain or no pain) was the outcome for the logistic regression analysis, and pain intensity was the outcome for negative binomial log regression using a conceptual pain model adapted for the study (Figure 1).^19^ First, bivariate analyzes were performed between the outcome and the independent variables chosen for the study, and later, regression models were constructed in a hierarchical approach, considering the adjustment of each block, in which: block 1 referred to the demographic and socioeconomic variables, size of the city and health system (which are more distal); block 2 counted on the use of the health service (health behavior) and the presence of cavities, which is a more proximal clinical variable. For the adjustment between the blocks, the cutoff point was p<0.20.

The chi-square test verified the association between school absenteeism and pain. A 5% significance level was adopted.

## RESULTS

Two large cities (L; n = 398), four medium-sized cities (M; n = 663) and two small cities (S; n = 180) were identified. The sample consisted of 1,241 children aged 12 years old, who were examined. From these, 1,233 returned the questionnaire answered by the parents. Therefore, for the study, 1,233 children were considered. Among the children examined, 16.7% (n = 206) reported pain in the last six months, with a prevalence of 15.2, 22.0 and 23.9%, respectively, for L, M and S cities. The sociodemographic data and the use of dental services are found in Table 1.

**Table 1:**
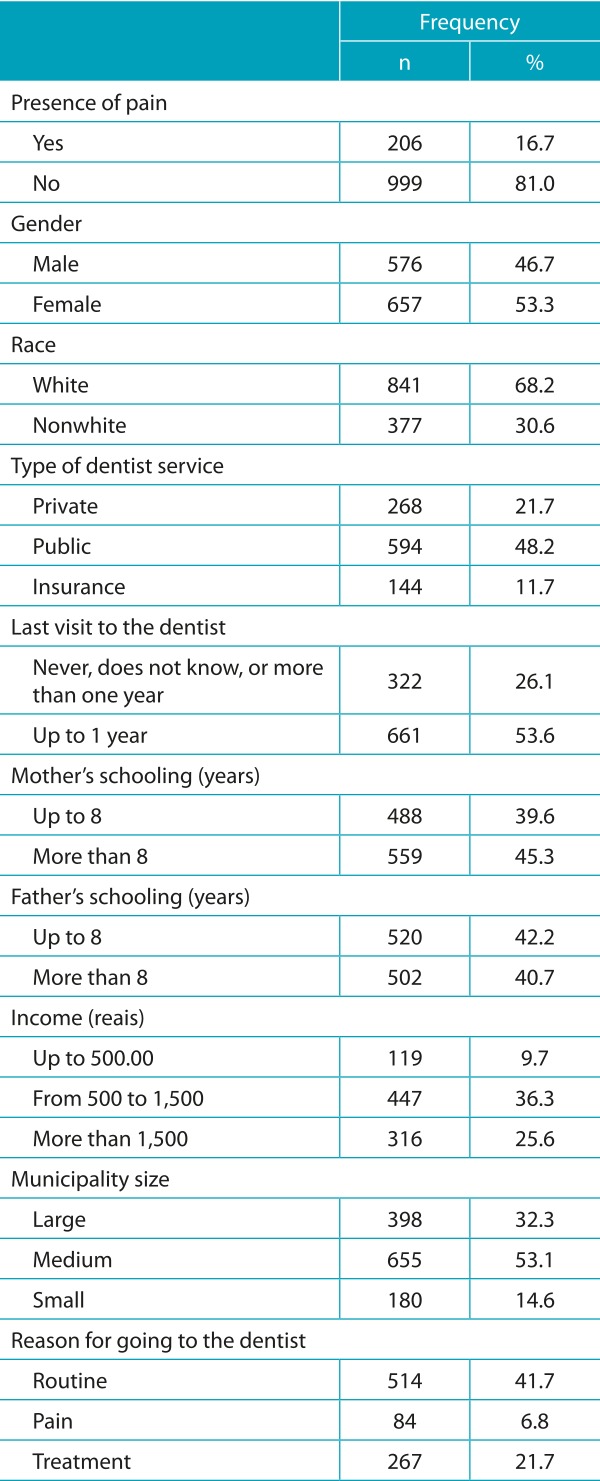
Demographic and socioeconomic characteristics, and the use of dental services of the sample. Regional Health Board VII of São Paulo, 2011.

The n of each variable does not match due to data loss caused by the lack of responses to the questionnaire. The total number of children who reported never having gone to the dentist was 46.

The distribution of variables occurred according to the prevalence (Table 2) and intensity (Table 3) of pain. Factors associated with the prevalence of dental pain were: self-reported race and family income. Nonwhites had higher prevalence in comparison to whites, whereas those with higher income (higher than R$ 1,500.00) had less chances of experiencing dental pain.

**Table 2:**
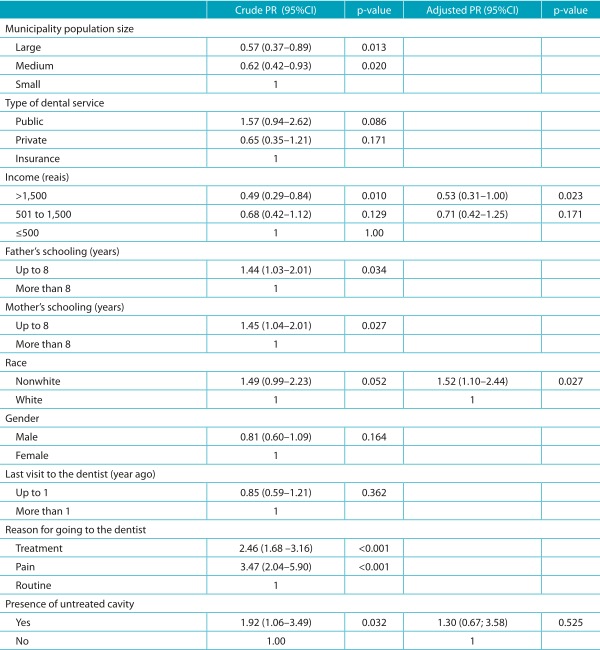
Demographic and socioeconomic variables associated with the prevalence of dental pain. Regional Health Board VII of São Paulo, 2011.

PR: Prevalence ratio. A simple logistic regression was performed for crude PR and a multiple logistic regression with a hierarchical approach was performed for adjusted PR.

**Table 3:**
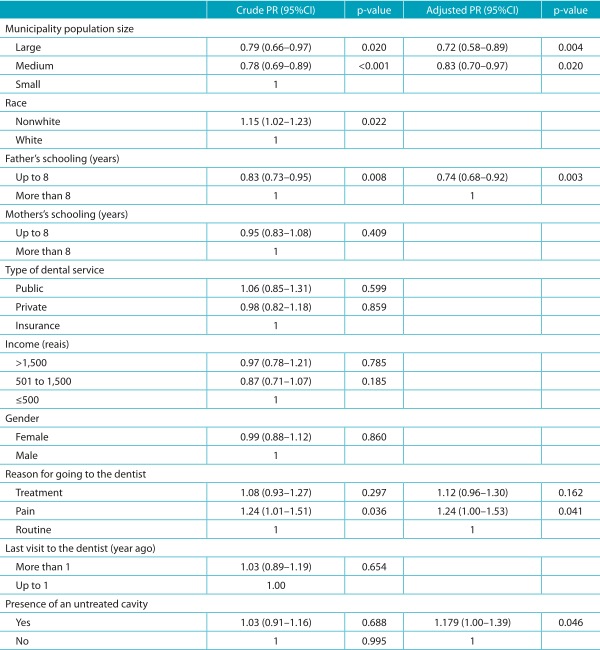
Demographic and socioeconomic variables associated with the intensity of dental pain. Regional Health Board VII of São Paulo, 2011.

A simple logistic regression was performed for crude PR and a multiple logistic regression with a hierarchical approach was performed for adjusted PR.

The average pain intensity reported was 4.97 ± 2.82, 4.33 ± 2.18 and 4.08 ± 2.06, respectively, for L, M and S cities. The size of the municipality, parental schooling and the treatment of cavities were the factors associated with the intensity of dental pain. The intensity of the pain decreased according to the size of the city. Regarding parental schooling, the results indicate that the lower the schooling, the greater the pain intensity reported by children. However, the variables “visit to the dentist due to pain” and “presence of teeth with school absenteeism is associated with reports of dental pain. Dental pain was associated with absenteeism among 84 schoolchildren who reported having missed school in the last six months.

**Table 4:**
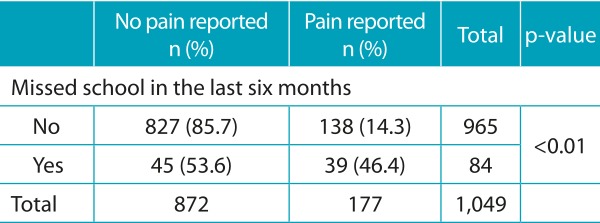
Dental pain and absenteeism of schoolchildren aged 12 years in the last six months. Regional Health Board VII of São Paulo, 2011.

Chi square test.

## DISCUSSION

Dental pain was associated with school absenteeism at the age of 12 and was less prevalent among students from high-income families, with higher prevalence among nonwhites. The intensity of dental pain was lower in small and medium-sized municipalities and higher among children who had not undergone dental treatment and who sought emergency services because of this pain.

Almost half (46.4%) of the children who missed classes reported that the absences were motivated by dental pain. Of the 1,233 children, 16.7% reported dental pain and, among those who answered questions about absenteeism and felt pain, 22.0% were absent from school due to tooth pain; 78.0% did not miss school, even with dental pain. The relationship between dental pain and school absenteeism was also verified in the study by Blumenshine et al.,^11^ who found that children with poor oral and general health conditions were 2.3 times more likely to have worse school performance. Piovesan et al.^12^ observed that oral problems increase the rate of school absenteeism, and that academic performance of children with lower socioeconomic level was impaired. Thus, it is suggested that improving the oral health conditions of children could also improve school performance.

The prevalence of dental pain found in the present study is within the range found in research in developed countries^5^ ^,^ ^6^ and in Brazil,^7^ ^,^ ^9^ which draws attention because pain has a negative impact on the quality of life of these children.^10^ ^,^ ^12^ However, these percentages cannot necessarily be generalized to represent the results of the prevalence of tooth pain in children in other regions of Brazil due to the existing socioeconomic differences. It is believed that the high Human Development Index (HDI) of these municipalities in the Campinas region influenced the health conditions found. A high prevalence of dental pain in children has been observed in other studies. In Sobral, Ceará, the prevalence of dental pain in children aged 11 to 15 years old was 31.8%.^21^ In Florianópolis, Santa Catarina, the prevalence of dental pain in schoolchildren aged 12 to 13 years was 33.7%.^22^ In a study conducted in Paulínia, São Paulo, the prevalence of dental pain in schoolchildren aged 12 to 13 years was 22.8% .^23.^

In an epidemiological survey performed in the state of São Paulo in 1998, a tendency for larger municipalities to present a lower DMFT index was observed.^24^ A similar observation was made by a study performed by Baldani et al.^25^ in Paraná, which registered that most small municipalities have high and very high cavity prevalence patterns, compared to large municipalities. In the city of São Paulo, Peres et al.^26^ concluded that dental pain was 33% less prevalent in adolescents living in more developed areas than among those living in less developed areas. This data does not corroborate with the findings of the present study, because the children of medium and small municipalities had lower average pain intensity. It is interesting to note that, even if feeling pain, some children did not go to the dentist. This fact may have occurred due to difficulties in accessing dental services, and there may be, in the specific case of these municipalities, more attention to oral health in smaller municipalities; or, because of the smaller population, the service can meet the demand better.

Having low HDI, being female, black, with low family income, having untreated cavities and the need for endodontic treatment were factors associated with dental pain in other studies.^25^ ^,^ ^26^ ^,^ ^27^ This data highlights the social inequalities in Brazil, which demonstrates that the population presents different health profiles, with worse indicators prevailing for groups with worse living conditions. In the present study, dental pain was also associated with parental low schooling, being nonwhite, “pain” as the reason for the search for dental services and untreated carious lesions. Higher income appeared as a protective factor for this type of pain.

The association between dental pain, income, and parental schooling observed in this study is well established in the literature. A study conducted in Belo Horizonte with 8 and 9 year-old students concluded there is greater prevalence and severity of toothache in children whose mothers have low schooling and socioeconomic disadvantage.^2^ In a study carried out in Florianópolis, Santa Catarina, Nomura et al. Al.^22^ demonstrated that children whose mother had low schooling were 2.5 times more likely to have dental pain, compared to those whose mothers’ educational level was higher. This data is explained by the possibility of greater discernment regarding the real need for treatment of the child when the mother has a higher level of schooling. Nomura et al.^22^ also found that children with a family income of up to US$ 67.00 were 3.2 times more likely to have dental pain, compared to children with a higher family income. Because of lower levels of parental education, family income is lower, which influences the living, working, employment and housing conditions. It is known that individuals of low socioeconomic level are more frequently exposed to several risk factors that affect their self-perception of oral health and well-being,^3^ ^,^ ^12^ and are more likely to have worse oral health conditions.^4^ ^,^ ^22^ ^,^ ^27^

Regular follow-up of the patient is essential for early diagnosis and immediate treatment of oral conditions in order to prevent them from worsening and being detrimental to quality of life.^27^ ^,^ ^28^ In this study, 26.1% of the children reported that their last visit to the dentist happened more than one year ago, or that they had never been to the dentist, which can be explained by their sporadic access to dental services, which is probably only available to treat emergencies, like it is in other age groups.^29^ The use of dental pain measurements enables a better assessment of the need for care and the establishment of priorities for oral health care,^28^ which can improve access to treatment. The importance of the Public Health System (SUS) in the use of dental services in the region was clear, considering that 48.2% of the children sought out public services. Thus, it is necessary to implement compensatory measures in the field of oral health promotion and dental care addressed at the most vulnerable population to limit the effects of social inequalities.^26^ ^,^ ^27^

Studies on dental pain using a contextual analysis are unusual, but may demonstrate that variables such as the population size of a municipality allow for a better understanding of the interaction between individual factors and those related to social environment. The multilevel analysis of factors associated with the prevalence and intensity of dental pain in the population studied was not performed, which may be considered as a study limitation. It is suggested that, for future studies, other contextual factors, besides the population size in the cities, should be included in a multilevel analysis with the other variables related to dental pain for the age of 12, as observed in the study Ferreira Júnior et al. with preschoolers.^13^

It was concluded that the prevalence and intensity of dental pain in 12 year-old children are related to socioeconomic aspects of the family, such as low income and low levels of parental schooling, which impact daily activity through school absenteeism. The intensity was lower in medium and small municipalities. It should also be pointed out that the children with greater intensity of dental pain were treated in emergency services. Considering this situation, the municipalities of this region should allocate resources for strategies to promote oral health that prevent the development of oral diseases, so that they do not reach the point of producing pain.
